# Epicatechin Gallate Regulation of Steroid Hormone Levels Improves Sarcopenia in C57BL/6J Mice

**DOI:** 10.3390/foods14142495

**Published:** 2025-07-16

**Authors:** Ziwei Huang, Meifeng Liu, Yufei Zhou, Yiyu Tang, Jian’an Huang, Sheng Zhang, Zhonghua Liu, Ailing Liu

**Affiliations:** 1 College of Bioscience and Biotechnology, Hunan Agricultural University, Changsha 410128, China; 17707921874@163.com; 2 National Research Center of Engineering and Technology for Utilization of Botanical Functional Ingredients, Changsha 410128, China; sx20230493@stu.hunau.edu.cn (M.L.); zhouyufei0723@163.com (Y.Z.); tangeu_1028@163.com (Y.T.); jian7513@sina.com (J.H.); liailing@hunan.cn (S.Z.); liuzhonghua@hunau.edu.cn (Z.L.); 3 Key Laboratory of Tea Science of Ministry of Education, Hunan Agricultural University, Changsha 410128, China; 4 Yuelushan Laboratory, Changsha 410128, China

**Keywords:** epicatechin gallate, steroid hormone, skeletal muscle, sarcopenia

## Abstract

The decline in differentiation capacity during skeletal muscle (SkM) aging contributes to the deterioration of skeletal muscle function and impairs regenerative ability. Epicatechin gallate (ECG), a major functional component of catechins found in tea, has an unclear role in aging-related sarcopenia. In vivo experiments in 54-week-old C57BL/6J mice showed that ECG treatment improved exercise performance, muscle mass, and fiber morphology and downregulated the expression of the testosterone metabolic enzyme gene *UGT2A3* in aged mice. In vitro experiments with Leydig cells (TM3) demonstrated that ECG upregulated the mRNA and protein expression levels of testosterone synthase genes, including *StAR*, *P450scc*, *3β-HSD*, *CYP17a1*, and *17β-HSD*. Network pharmacology analysis further suggested that ECG can influence testosterone secretion through the regulation of cytokines, thereby promoting skeletal muscle differentiation. These findings indicate that ECG enhances the differentiation of skeletal muscle cells by modulating testosterone levels, which helps alleviate age-related muscle function decline.

## 1. Introduction

Sarcopenia is defined as a decrease in skeletal muscle mass, strength, or function. The most prevalent form of muscle atrophy in humans is the muscle loss associated with aging, which is characterized by a reduction in muscle mass and strength, as well as diminished mobility [1]. The area and number of muscle fibers will decrease with age [2,3]. Additionally, aging may disrupt the interaction between myosatellite cells and their surrounding cells, leading to impaired muscle regeneration [4]. At this stage, numerous studies have demonstrated that various natural polyphenols can effectively treat skeletal muscle diseases. The study by Chen et al. [5] indicated that tea polyphenols can alleviate symptoms of muscle atrophy in mice through the mechanism of mitochondrial quality control. Tea polyphenols can reduce apoptosis of skeletal muscle cells and improve exercise ability [6]. Polyphenols can activate the Kelch-like ECH-associated protein 1- Nuclear factor erythroid 2-related factor 2 (Keap1-Nrf2) signaling pathway, significantly improving the activity of antioxidant enzymes and the expression of antioxidant genes in mice [7]. Human experiments have shown that polyphenols may have a positive impact on muscle damage through pathways such as regulating inflammatory factors [8].

Endocrine hormones play a crucial role in regulating skeletal muscle metabolism. They help stabilize the balance between the synthesis and degradation of myocyte proteins, thereby maintaining skeletal muscle mass and function. The reduction in the secretion of various endocrine hormones such as growth hormone, sex hormones, and insulin is a direct cause of skeletal muscle atrophy. Although the mechanisms underlying sarcopenia are not yet fully understood, some studies suggest that a reduction in androgen levels is one of the contributing factors [9]. Testosterone (T) is a steroid hormone secreted by the male testes or female ovaries. It helps maintain muscle strength and mass, supports bone density and strength, and can also boost energy and enhance physical performance. Testosterone is the primary androgen in males and can promote muscle regeneration and repair by increasing the number of muscle satellite cells [10]. Testosterone promotes the expression of insulin-like growth factor-1 (IGF-I) in muscles, thereby activating the Akt-mTOR signaling pathway, which induces muscle hypertrophy [11]. Testosterone can directly stimulate the Ras/mitogen-activated extracellular signal-regulated kinase/extracellular signal-regulated kinase (Ras/MEK/ERK) pathway in muscle cells, inhibiting the expression of myostatin [12]. Testosterone also has a strong anti-apoptotic effect in muscle, leading to the inactivation of Forkhead box O (FOXO) and inhibiting the upregulation of pro-apoptotic genes [13]. Numerous studies have shown that many natural polyphenolic substances can promote the secretion of steroid hormones. Daidzein can regulate the expression of genes such as *StAR*, *P450scc*, and *3β-HSD*, promoting the synthesis of steroid hormones in granulosa cells [14]. Cheng et al. [15] found in their study on piglets that soy isoflavones can upregulate the serum testosterone levels.

Tea has a long history in China. Drinking tea not only refreshes the mind but also promotes health and wellness, making it highly popular among people. Catechins are the main components of tea polyphenols. Research shows that catechins have pharmacological effects such as scavenging free radicals in the body as well as anti-aging and anti-radiation effects. The beneficial properties of catechins on skeletal muscle are mainly reflected in enhancing SkM satellite cell differentiation, promoting muscle mitochondrial synthesis, enhancing capillary biogenesis, and combating age-related SkM degeneration [16]. Catechins promote myoblast differentiation by stimulating the myogenic signaling pathway and the p38 mitogen-activated protein kinase (p38MAPK)/protein kinase B (AKT) pathway. Catechin can also positively regulate *MyoD*-mediated muscle-specific gene expression (such as *MyHC* and *MyoG*), increasing the conversion of fibroblasts [17]. To further explore the role of catechins in promoting skeletal muscle differentiation and their molecular mechanisms, the research team previously used four different catechins to culture C2C12 cells; measured the relative expression levels of muscle differentiation markers *MyoD*, *MyoG*, and *MyHC* mRNAs; and assessed the number, length, and diameter of differentiated myotubes. The results indicated that epicatechin gallate (ECG) had a stronger pro-differentiation effect than other catechin monomers, and its pro-differentiation effect increased with concentration [18]. The pro-differentiation effect of ECG suggests that it may have the potential to improve sarcopenia.

To investigate the impact and mechanism of ECG on the motor function and differentiation of skeletal muscle in aged mice, this study, based on previous research, utilized 54-week-old C57BL/6J mice as the research subjects. Animal behavioral experiments were conducted to assess changes in motor ability across different age groups of mice. Tissue sectioning and transcriptomic analysis were employed to preliminarily analyze the mechanistic effects of ECG on sarcopenia of aging. Additionally, this study verified the effect of ECG on steroid hormones using the TM3 cell model. It was found that ECG influenced the gene expression of testosterone synthetase, as demonstrated using qRT-PCR and Western blot experiments. Furthermore, the potential target of ECG in affecting skeletal muscle differentiation was predicted using network pharmacology. In summary, our study is the first to demonstrate that ECG can influence the function and differentiation of skeletal muscle in aged mice by regulating the synthesis and metabolism of testosterone, providing a theoretical basis for ECG as a potential dietary supplement for the protection of aging skeletal muscle.

## 2. Materials and Methods

### 2.1. Experimental Materials and Animals

ECG (purity 98%) was purchased from Hunan Sunfull Bio-tech Co., Ltd. (Changsha, China).

Sixty healthy male C57BL/6J mice of different ages were provided by Jicui Pharmaceutical Biotechnology Co., Ltd. (Nanjing, China). All animals were acclimatized for one week, with a rearing environment temperature of 25 ± 1 °C, relative humidity of 50 ± 1%, and a light/dark cycle of 12/12 h. All animal experiments were conducted in accordance with the animal husbandry regulations of Hunan Agricultural University (Approval No. 2025 No. 4). The subjects were divided into several groups: the juvenile control group (Y-C) consisting of 10 mice aged 3 weeks, the juvenile ECG group (Y-ECG) with 10 mice also aged 3 weeks, the middle-aged control group (M-C) comprising 10 mice aged 26 weeks, the middle-aged ECG group (M-ECG) with 10 mice aged 26 weeks, the elderly control group (O-C) containing 10 mice aged 54 weeks, and the elderly ECG group (O-ECG) with 10 mice aged 54 weeks.

According to the “Scientific opinion on the safety of green tea catechins” published by the European Food Safety Authority (EFSA) [19], it is pointed out that the daily intake of EGCG in human body is safe under 800 mg. According to the dose conversion between human and experimental animals [20], the maximum dose of ECG was 160 mg/kg/d (800 mg/60 kg/d ∗ 12.3 = 164 mg/kg/day). Following the grouping, the control group received a normal diet supplemented with gavage drinking water (160 mg/kg/d), whereas the experimental group was given a normal diet supplemented with gavage ECG (160 mg/kg/d). The experiment was conducted over a period of 8 weeks, during which the initial weight of the mice was recorded at the start and subsequently measured weekly. Animal behavioral tests were conducted after the completion of the gavage treatment.

### 2.2. Motor Ability Test of Mice

The grip strength of the mice was assessed using an electronic grip tester (Shanghai, China). The grip meter platform was positioned horizontally to allow the mice’s limbs to grasp the sensing rod of the grip meter. The mouse’s tail was then pulled horizontally backward until its forelimbs released the sensing rod. This procedure was repeated five times to collect measurement data. The maximum value of the median grip strength was recorded as the absolute grip of the mice (N). The relative grip (N/g) was calculated by dividing the absolute grip by the weight of the mouse (N).

An animal treadmill (Huaibei, China) was used for testing. The animal treadmill has a total of 8 lanes for mice to undergo exercise endurance tests. To achieve optimal results, varying speeds were set for different time segments: 0 to 5 min at 10 m/min, 5 to 10 min at 15 m/min, 10 to 15 min at 20 m/min, and 15 to 20 min at 25 m/min. This approach allows the animals to quickly acclimate to the training rhythm. Each training session lasted 20 min and was conducted once daily, with a total of three training sessions. During the formal test, the speed for the initial 5 min was set at 10 m/min, followed by an increase of 5 m/min every subsequent 5 min until the mice reached exhaustion. The speed at exhaustion (m/min) and the total distance traveled (m) were recorded.

Testing was conducted using a mouse rotarod apparatus (Shanghai, China). The instrument has 6 rotating rods for conducting experiments with mice. Mice were placed on the rotary rod, where the rotation speed was initially set to 10 r/min for 1 min, followed by a 5-min training period. Subsequently, the speed was increased to 20 r/min for an additional 5 min. During the initial training phase, the experimenter assisted the mice by gently grasping their tails to encourage movement in the opposite direction of the rotating rod. Once the mice stabilized, they were allowed to crawl freely. The timing commenced when a mouse fell due to errors or fatigue. Prior to the formal experimentation, the mice underwent a training period lasting 3 days. During the actual test, timing began at 4 r/min for 1 min, with a uniform acceleration from 4 r/min to 40 r/min over a duration of 10 min. The time until each mouse fell was meticulously recorded.

### 2.3. Animal Euthanasia and Tissue Collection

An intraperitoneal injection was administered using 2% sodium pentobarbital at a dosage of 40 mg/kg. Once the mice were fully anesthetized, blood was collected from the mouse’s eye into a 2 mL centrifuge tube, which was then stored at 4 °C for 1 h. Following this, the samples were centrifuged at 3000 r/min for 20 min to collect the supernatant, which was subsequently stored at −80 °C. After blood collection, the mice were positioned supine on a plate, and the chest cavity was exposed. A perfusion needle was inserted into the aorta, and the right atrium was quickly incised. Pre-cooled saline was then perfused until the liver appeared white and the effluent was clear, indicating that the perfusion was complete. Intact skeletal muscles from both sides of the mice were isolated on ice, and their wet weight (g) was measured using an analytical balance. Following this, a portion of the mice was fixed in 10% neutral formalin, and paraffin-embedded sections were prepared for subsequent pathological analysis. The tissues from the remaining mice were wrapped in tin foil, immediately placed in a liquid nitrogen tank, and subsequently transferred to −80 °C for storage in the refrigerator to facilitate future nucleic acid detection.

### 2.4. Organ Index

Following blood collection, each mouse was euthanized via cervical dislocation, and the spleen, thymus, heart, kidneys, and liver were excised. Excess adipose tissue was carefully removed, and the organs were rinsed with saline to eliminate surface blood. The organs were then gently blotted with filter paper to remove any excess moisture and weighed, and the organ index was calculated. The formula for calculating the organ index is as follows:

Organ index = Organ weight (g)/Body weight (g) × 100%

### 2.5. Muscle Histological Staining

Samples from the gastrocnemius muscle were fixed in 10% neutral buffered formalin (for at least 24 h) and transversely trimmed. After paraffin embedding using an automatic tissue processor (Wuhan, China) and embedding machine (Wuhan, China), nine continuous 5 μm sections were prepared using a microtome (Shanghai, China). Histopathological staining was performed with hematoxylin and eosin (H&E) (Wuhan, China). Detection was then carried out using an optical microscope (Tokyo, Japan) and an automatic image analyzer (Tokyo, Japan).

Staining was performed using the following method: take 1 mm^3^ of muscle tissue and place it in 2.5% glutaraldehyde fixative (Wuhan, China) at 4 °C for 24 h; rinse the tissue with 1×PBS (Wuhan, China) at 4 °C three times, 15 min each time; fix with 1% osmium tetroxide (Wuhan, China) at 4 °C overnight; rinse the tissue with 1× PBS at 4 °C three times, 15 min each time; stain with 2% uranyl acetate solution (Wuhan, China) for 2 h. After gradient dehydration with ethanol solutions (Wuhan, China) of different concentrations (30%, 50%, 70%, 80%, 90%, 95%, 100%), dehydration was performed with 100% acetone solution (containing anhydrous copper sulfate) (Wuhan, China) for 30 min. Embedding was performed with epoxy resin at 37 °C for 12 h. Trim the resin, cut thin sections at room temperature, control the thickness to 50–100 nm, and fix the tissue sections on copper grids. Next, stain with uranyl acetate in the dark for 30 min, rinse with deionized water, restain with lead citrate in the dark for 15 min, and rinse with deionized water. After drying, observe the ultrastructure of the tissue using TEM (Hillsboro, OR, USA).

The cross-sectional area (CSA) of myofibers and the quantity of muscle fibers were determined using ImageJ v.1.54g analysis software (Bethesda, MD, USA), and the average cross-sectional area of myofibers was subsequently calculated. The measurement method involved taking the same field of view for each slice while ensuring that the background lighting of each photograph remained consistent. ImageJ software was then utilized to analyze the images and obtain all relevant measurement data.

### 2.6. qRT-PCR Detects Differentiation-Related Genes

Gastrocnemius tissues from both the OC and O-ECG groups were extracted, and the mRNA levels of *MyoD*, *MyoG*, *MRF4*, and *MCK* were assessed using the Aikore qPCR kit (Changsha, China) for RNA extraction, reverse transcription, and real-time quantitative PCR. The relative expression levels were then analyzed.

### 2.7. Transcriptome Sequencing and Analysis

Randomly select gastrocnemius muscle tissue samples from mice in the O-C group and the O-ECG group, and commission Gidi BioTech Co., Ltd. (Guangzhou, China) to complete cDNA library construction, sequencing, and analysis. Using *p* < 0.05 and |log2FC| > 1.5 as criteria, differentially expressed genes (DEGs) were screened from the two groups, and then GO and KEGG enrichment analyses were performed on the screened DEGs.

### 2.8. Cellular Experiment Verification

The cell experiment was conducted by Shanghai Anwei Biotechnology Co., Ltd. (Shanghai, China). Testicular interstitial cells from mice (TM3) in the logarithmic growth phase were digested using trypsin and then prepared into a suspension at a concentration of 1 × 10^5^ cells/mL. These cells were subsequently seeded into 96-well plates at a density of 1 × 10^4^ cells per well. Following this, 100 μL of culture medium was added to each well, and the plates were placed in a CO_2_ incubator (5%) at 37 °C for 24 h, after which they were cultured without serum for an additional 24 h. After culturing, half of the cell sample culture medium was replaced with ECG at concentrations of 1, 5, 25, 50, 100, and 200 μg/mL for 24 h. The control group received a solvent-containing medium. Following the incubation period, CCK-8 solution was added to all wells, and the samples were incubated for an additional 2 h. The absorbance at 450 nm was measured using a microplate reader (Wuxi, China). The cell survival rate was calculated using the following formula: Survival rate% = (experimental group − blank group)/(control group − blank group).

### 2.9. Testosterone Concentration Measurement

The testosterone concentration in the supernatant of the cell culture medium was measured after 24 h using a Mouse Testo ELISA Kit (Wuhan, China). The analysis included a control group and an ECG treatment group (ECG 50 μm/mL). Absorbance values for each well were determined using a microplate reader (Shanghai, China) at a wavelength of 450 nm. The gastrocnemius muscle tissues from both the O-C group and the O-ECG group were collected, and the testosterone concentration was measured using the BCA Protein Quantification Kit (Changsha, China) and the Mouse Testo ELISA Kit (Wuhan, China).

### 2.10. qRT-PCR Detection

TM3 cells in the logarithmic growth phase were harvested and seeded into 6-well plates at a density of 50 × 10^4^ cells per well. Once the cell density reached 70%, 2 mL of DMEM/F12 containing 1% antibody was added to each well in the control group. In the ECG treatment group, 2 mL of 50 μg/mL ECG was added per well, and the cells were treated for 24 h. Subsequently, cellular RNA was extracted, and reverse transcription followed by real-time quantitative PCR was performed to assess the mRNA expression levels of testosterone synthetase genes, including *StAR*, *P450scc*, *3β–HSD*, *CYP17a1*, and *17β–HSD*. Gastrocnemius tissues from both the OC group and the O-ECG group were collected, and the relative expression level of the testosterone-metabolizing enzyme gene *UGT2A3* was assessed using the Aikore qPCR kit (Changsha, China) for RNA extraction, reverse transcription, and real-time quantitative PCR. Primers were designed and synthesized by Shenggong Bioengineering Co., Ltd. (Shanghai, China), with all primer sequences listed in Table S1. The *β-actin* gene served as the internal reference gene, and three replicates were conducted for each group. The relative expression levels of each gene were calculated using the 2^−ΔΔCt^ method.

### 2.11. Western Blot Detects Protein Expression Levels

Cultivate TM3 cells with 50 μg/mL ECG for 24 h, extract total cellular protein, and determine the sample protein concentration using the BCA protein concentration assay kit (Chengdu, China). According to the measured concentration, take the corresponding volume of sample to achieve 40 μg of protein per well, add 5× protein loading buffer, and incubate in a boiling water bath at 95–100 °C for 5 min. The following steps are performed: gel preparation, sample loading, electrophoresis, transfer, blocking for 1 h, incubation with primary antibody at 4 °C overnight, incubation with secondary antibody at room temperature for 30 min, ECL reaction, and darkroom exposure. Finally, the film is scanned and archived, and the AlphaEaseFC v.4.0 software processing system analyzes the optical density values of the target bands. Detect the protein expression levels of the testosterone synthesis enzyme genes *StAR*, *P450scc*, *3β-HSD*, *CYP17a1*, and *17β-HSD*, using *β-actin* as an internal reference.

### 2.12. Network Pharmacology Target Prediction and Validation

To conduct the analysis, enter the keywords “Epigallocatechin gallate,” “Testosterone,” or their structures into the TargetNet, CTD, TCMSP, Swiss Target Prediction, and Pharm Mapper databases. Additionally, input “Sarcopenia” into the Genecards, Drugbank, and CTD databases. After intersecting the target data, the effects of epigallocatechin gallate on potential targets related to sarcopenia through modulation of testosterone levels were assessed. The Venn diagram and protein–protein interaction (PPI) network were generated using Venny, String database tools, and Cytoscape version 3.10.2.

To validate the predicted results of network pharmacology targets, serum samples from the O-C group and the O-ECG group were taken and measured using Tumor Necrosis Factor-α (TNF-α) and Interleukin-6 (IL-6) detection kits (Wuhan, China). The concentration of the standard product was plotted on the vertical axis, while the optical density value was plotted on the horizontal axis. The professional curve production software “Curve Expert v.1.4” was employed for analysis, and standard curves were generated. The regression equation of the standard curve was calculated based on the concentration and OD values of the standard product. Subsequently, the OD values of the samples were substituted into the equation to determine the sample concentrations.

### 2.13. Statistical Analysis

SPSS 27.0.1 software (Armonk City, NY, USA) and Graphpad Prism 10.1.2 (San Diego City, CA, USA) were used for statistical analysis, and relevant data are expressed as mean ± standard deviation (x% ± s). An independent sample test was used for comparison between groups, and a non-parametric test was used if it did not conform to the normal distribution. For this study, *p* < 0.05 was considered statistically significant.

## 3. Results

### 3.1. ECG Can Enhance Motor Performance in Mice

**Figure 1:**
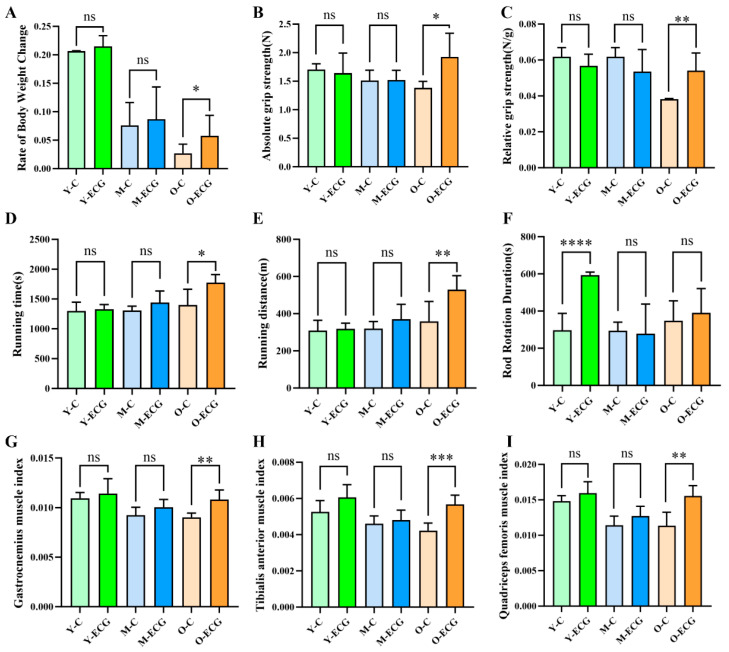
ECG improves weight loss and enhances skeletal muscle capacity in aged mice. (**A**) Changes in body weight of the mice. (**B**) Absolute grip strength of the mice. (**C**) Relative grip strength of the mice. (**D**,**E**) Running time and distance on the treadmill. (**F**) Rotarod running time of the mice. (**G**–**I**) Skeletal muscle index of the mice. Data are presented as mean ± SD (*n* = 3–9). Compared with the control group, * *p* < 0.05, ** *p* < 0.01, *** *p* < 0.001, **** *p* < 0.0001, ns > 0.05.

After 8 weeks of ECG intervention, the body weight changes in mice demonstrated significant age-dependent variations (Figure 1A). Compared to their respective control groups, ECG treatment significantly promoted body weight gain, with this effect becoming more pronounced with advancing age. Specifically, the Y-ECG group showed a weight gain rate of 0.215 ± 0.019%, representing a 3.9% increase compared to the Y-C group (0.207 ± 0.001%). The M-ECG group exhibited a weight change rate of 0.087 ± 0.057%, which was 14.5% higher than that of the M-C group (0.076 ± 0.040%). Most notably, the O-ECG group displayed the most significant alterations, with a weight change rate of 0.058 ± 0.036% (*p* = 0.0410)—a remarkable 114.8% increase relative to the O-C group (0.027 ± 0.016%). These results indicate that ECG intervention exerts a more pronounced promoting effect on body weight regulation in aged mice. Following the completion of the gavage, the muscle capabilities of the mice were assessed. As demonstrated in our results, the O-ECG group exhibited significantly enhanced grip strength compared to the O-C group. The absolute grip strength of the O-ECG group was 1.93 ± 0.42 g (*p* = 0.0118), which was significantly higher than that of the O-C group (1.39 ± 0.11 g). Similarly, the relative grip strength in the O-ECG group increased significantly to 0.054 ± 0.010 (*p* = 0.0029) compared to 0.038 ± 0.001 in the O-C group. However, ECG had no significant effect on the absolute and relative grip strength of juvenile and middle-aged mice (Figure 1B,C). The O-ECG group exhibited a significant increase in treadmill endurance capacity, with a running time of 1774 ± 135 s (*p* = 0.0113)—a 26.6% improvement over the O-C group (1401 ± 263 s). Concomitantly, the total running distance in the O-ECG group reached 530 ± 75 m (*p* = 0.0093), demonstrating a 47.6% enhancement compared to 359 ± 106 m in the O-C group (359 ± 106 m). Notably, no significant differences in running performance were observed between ECG-treated and control groups in either young or middle-aged mice (Figure 1D,E). The rotarod performance time in the aged group showed no significant difference, whereas the motor coordination of juvenile mice was significantly improved (*p* = 0.001) (Figure 1F).

The assessment of muscle mass is quantified by the skeletal muscle index, defined as the ratio of muscle mass to body weight. Statistical analysis revealed that the gastrocnemius muscle index in the O-ECG group mice was 0.01082 ± 0.00098 (*p* = 0.0022), demonstrating a significant increase compared to 0.00902 ± 0.00043 in the O-C group (Figure 1G). Similarly, the tibialis anterior muscle index in the O-ECG group was 0.00568 ± 0.00051 (*p* = 0.0003), which was significantly higher than 0.00422 ± 0.00042 in the O-C group (Figure 1H). The quadriceps femoris muscle index in the O-ECG group was 0.01555 ± 0.00146 (*p* = 0.0016), showing a notable elevation compared to 0.01136 ± 0.00189 in the O-C group (Figure 1I). No significant changes in muscle indices were observed in juvenile and middle-aged mice.

### 3.2. ECG Can Enhance the Morphological Development of Skeletal Muscle in Aging Mice and Upregulate the Expression of Genes Associated with Skeletal Muscle Differentiation

**Figure 2:**
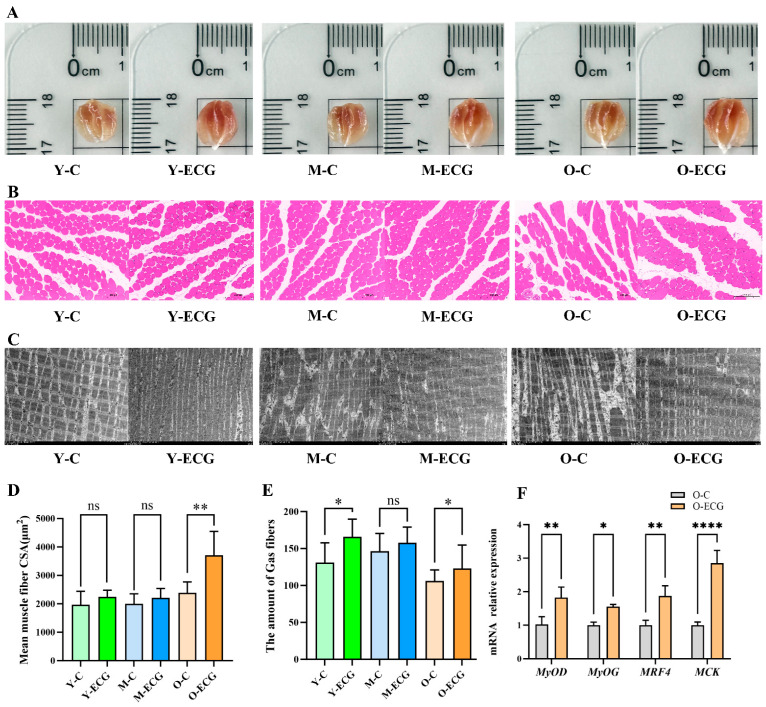
ECG significantly influences the morphology of skeletal muscle fibers in mice. (**A**) Samples of the gastrocnemius muscle from the mice are presented. (**B**) H&E staining illustrates the morphology of muscle fibers at 20× magnification. (**C**) Transmission electron microscopy is employed to observe the arrangement of muscle fibers in the mice. (**D**,**E**) ImageJ software was utilized for the statistical analysis of the average cross-sectional area and the number of muscle fibers in the mice. (**F**) The relative expression levels of mRNA for skeletal muscle differentiation-related genes in the mice are reported. Data are presented as mean ± standard deviation (*n* = 6). Data are expressed as mean ± standard deviation (n = 6). Compared to the control group, * *p* < 0.05, ** *p* < 0.01, **** *p* < 0.0001, ns > 0.05.

After the behavioral test, the mice were anesthetized and dissected, and the gastrocnemius muscles of the mice were photographed and weighed. It was found that the length and width of the fresh gastrocnemius muscles of young, middle-aged, and old mice treated with ECG were increased to varying degrees (Figure 2A). H&E staining showed that there was no significant difference in the shape and arrangement of muscle fibers between the Y-C and Y-ECG groups. There were no significant differences in the morphological distribution and number of muscle fibers between the M-C and M-ECG groups (Figure 2B). The muscle fibers in the O-C group showed irregular shape and different sizes, and the muscle fibers were loosely arranged with large gaps. The muscle fibers of mice in the O-ECG group were intact in shape, more numerous, and closely arranged (Figure 2B). The ultrastructure of skeletal muscle fibers of mice in different treatment groups was observed and photographed using transmission electron microscopy, and the results showed that the muscle fibers of mice in the Y-ECG group were relatively closely arranged and more numerous (Figure 2C). In the M-C group, muscle fibers were sparsely arranged and disorganized, which recovered slightly after ECG treatment (Figure 2C). The muscle fiber structure of the O-C group was completely disordered, and the thick and thin myofilaments were loosely arranged. ECG intervention could basically restore the arrangement and distribution of muscle fibers in the aging mice, and the myotome was arranged neatly and orderly (Figure 2C).

ImageJ software was used to analyze the data of the sections, and it was found that the myofiber area and the number of myofibers in the ECG-treated mice tended to increase. The mean cross-sectional area of muscle fibers in the O-ECG group was 3711.02 ± 840.87 μm^2^ (*p* = 0.0012), exhibiting a statistically significant increase compared to 2389.90 ± 378.25 μm^2^ in the O-C group (Figure 2D). The number of muscle fibers in the Y-ECG group was 194.17 ± 11.09 (*p* = 0.0392) (Figure 2B), showing a notable elevation compared to 94.67 ± 23.01 in the Y-C group, with a mean difference of 34.83 ± 14.69. Similarly, the O-ECG group demonstrated a higher muscle fiber count of 139.25 ± 25.00 (*p* = 0.0266) (Figure 2E) compared to 98.50 ± 12.37 in the O-C group, with an average difference of 40.75 ± 13.95. Based on the results, we concluded that ECG significantly impacts the exercise capacity and skeletal muscle mass of aged mice and notably ameliorates aging-induced skeletal muscle atrophy. Consequently, we selected aged mice for subsequent experimental studies to explore the mechanisms by which ECG influences skeletal muscle mass. qRT-PCR analysis of gastrocnemius muscles from aged mice revealed that the relative mRNA expression levels of skeletal muscle differentiation regulatory genes *MyoD*, *MyoG*, *MRF4*, and *MCK* in the O-ECG group were 1.826 ± 0.1901 (*p* = 0.0025), 1.558 ± 0.1901 (*p* = 0.0398), 1.870 ± 0.1901 (*p* = 0.0013), and 2.851 ± 0.1901 (*p* = 0.0001), respectively (Figure 2F). All these values showed statistically significant upregulation compared to the O-C group.

### 3.3. ECG Did Not Affect the Physical Condition of the Mice

By observing changes in organ weight, researchers can gain insights into the nutritional status of the body and the health condition of internal organs. After eight weeks of oral gavage with ECG, the effects of ECG on the organ index of mice are presented in Table S1.

### 3.4. ECG Can Modify the Expression of Genes in the Gastrocnemius Muscle of Aged Mice

**Figure 3:**
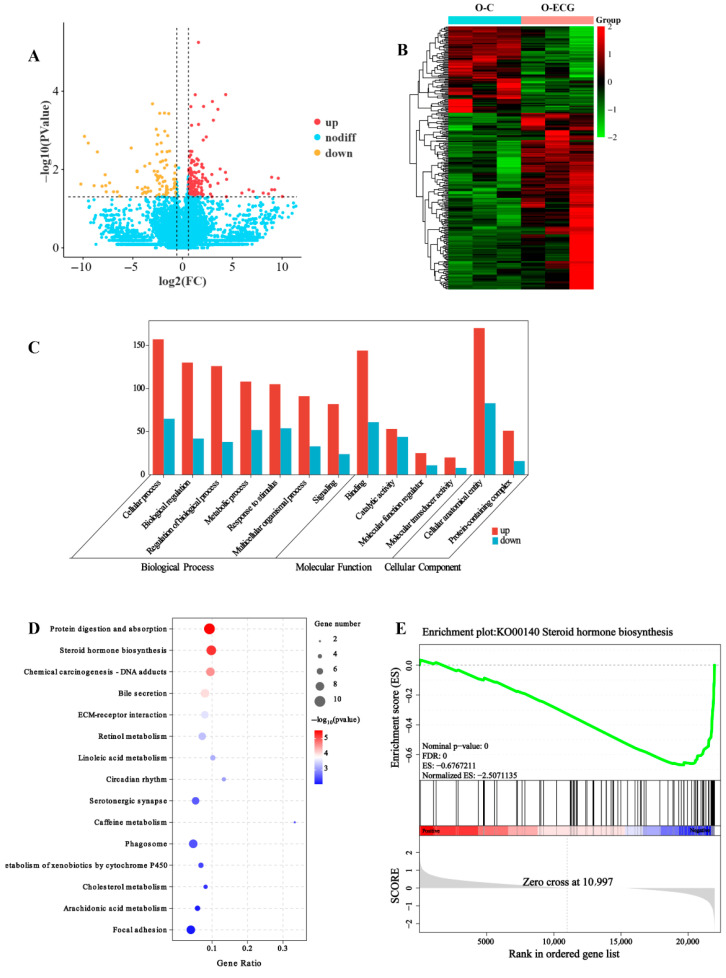
ECG-induced skeletal muscle differentiation in aged mice, differential gene expression profile, and bioinformatics analysis. (**A**) Volcano plot illustrating differentially expressed genes. (**B**) Heatmap displaying the clustering of differentially expressed genes. (**C**) GO pathway enrichment classification of the differentially expressed genes. (**D**) KEGG pathway enrichment classification of the differentially expressed genes. (**E**) GSEA_KEGG pathway enrichment analysis.

To further investigate the molecular mechanisms by which ECG improves sarcopenia, RNA-seq was employed to examine the gene expression profiles in the gastrocnemius muscle tissues of aged mice. Transcriptome sequencing provided transcriptomic data from both the O-C group and the O-ECG group. Following rigorous data screening, quality control, and preliminary analyses, the reliability of the transcriptome data was established. The differences in transcript data between the two groups were analyzed, with a threshold for gene expression differences set at ±1.5-fold, and a *p*-value < 0.05 was deemed significant. A total of 268 differential genes were identified (Figure 3A,B), including 180 upregulated and 88 downregulated DEGs.

The results of the Gene Ontology pathway enrichment analysis indicate that, in biological processes (Figure 3C), the differential gene enrichment is primarily associated with cellular processes, biological regulation, regulation of biological processes, metabolic processes, and responses to stimuli. In terms of molecular functions, the differential gene enrichment is related to linkage processes, catalytic activity, molecular function regulation, and molecular converter activity, among others. Regarding cellular components, the enrichment predominantly involves cell anatomy and protein-containing complexes.

To further elucidate the primary enrichment signaling pathways associated with differentially expressed genes, KEGG pathway enrichment analysis was conducted. The results indicated that ECG-induced differentially expressed genes were significantly enriched in skeletal muscle-related pathways, including ECM-receptor interaction, bile secretion, and steroid biosynthesis pathways (Figure 3D). Additionally, Gene Set Enrichment Analysis (GSEA) was performed on the differentially expressed genes to assess the distribution trends of metabolic pathways during pathway enrichment. The findings revealed that among the differential genes regulated by ECG, the steroid biosynthetic pathway was the most prominent, ranking first in significance (Figure 3E).

### 3.5. ECG Can Affect Testosterone Secretion

**Figure 4:**
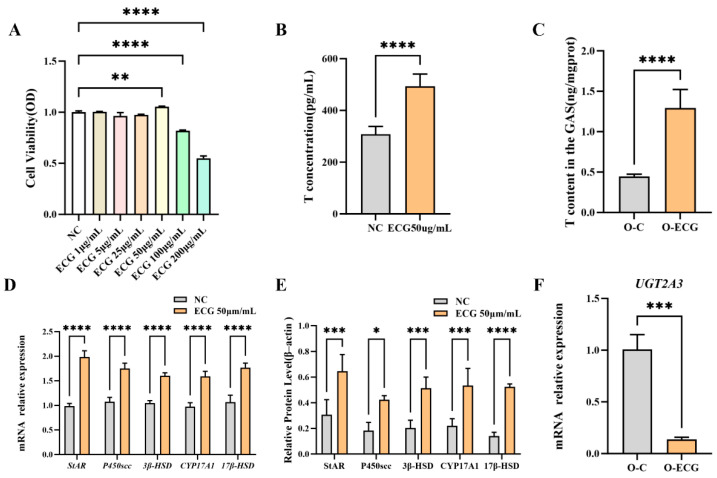
(**A**) TM3 cell viability. (**B**) Testosterone concentration in the supernatant of TM3 cells. (**C**) Testosterone content in the gastrocnemius muscle of mice. (**D**,**E**) Relative mRNA expression levels and protein expression levels of testosterone synthase genes. (**F**) Relative mRNA expression levels of testosterone metabolic enzyme genes. Data are presented as mean ± standard deviation (*n* = 3). Compared to the control group, * *p* < 0.05, ** *p* < 0.01, *** *p* < 0.001, **** *p* = 0.0001.

To verify the effect of ECG on promoting testosterone hormone synthesis, cell culture experiments were conducted. The experimental results indicate that after 24 h of treatment with ECG, the viability of TM3 cells in the 50 μg/mL treatment group was significantly higher than that of the control group (*p* = 0.0060). Conversely, the cell viability in the 100 μg/mL and 200 μg/mL treatment groups was significantly lower than that in the control group (*p* = 0.0001). These findings demonstrate that a 50 μg/mL concentration of ECG significantly promotes the proliferation of TM3 cells, while 100 and 200 μg/mL concentrations significantly inhibit their proliferation (Figure 4A). Consequently, a concentration of 50 μg/mL ECG was selected for further testing.

After 24 h of treatment with 50 μg/mL ECG, the supernatant from the TM3 cell culture medium was collected, and the testosterone concentration was measured using ELISA. The results indicated that the testosterone concentration in the supernatant of the test group was (493.9 ± 46.97) μg/mL, which was significantly higher than that of the control group, which measured (308.06 ± 30.42) μg/mL (*p* = 0.0001) (Figure 4B). Concurrently, testosterone content assays in gastrocnemius muscles of aged mice revealed that the O-ECG group exhibited a testosterone level of 1.2934 ± 0.2284 ng/mgprot (*p* = 0.0001), demonstrating a statistically significant 2.9-fold increase compared to 0.4467 ± 0.0277 ng/mgprot in the O-C group (Figure 4C). In order to explore the regulatory mechanism of ECG promoting testosterone synthesis, the relative mRNA expression levels of testosterone synthase genes *StAR*, *P450scc*, *3β-HSD*, *CYP17a1*, and *17β-HSD* in TM3 cells were detected using qRT-PCR. The results showed that, the relative expression levels of *StAR*, *P450scc*, *3β-HSD*, *CYP17a1*, and *17β-HSD* genes in TM3 cells cultured by ECG were 1.98 ± 0.13, 1.75 ± 0.11, 1.60 ± 0.06, 1.59 ± 0.10, and 1.77 ± 0.09, respectively. They were significantly greater than those in the control group (*p* = 0.0001) (Figure 4D). The protein expression levels of testosterone synthase StAR, P450scc, 3β-HSD, CYP17a1, and 17β-HSD in TM3 cells were detected by Western blot. Protein expression levels of testosterone biosynthesis enzymes in the control group of TM3 cells were as follows: StAR, 0.31 ± 0.12; P450scc, 0.18 ± 0.06; 3β–HSD, 0.20 ± 0.06; CYP17a1, 0.22 ± 0.06; and 17β–HSD, 0.14 ± 0.03. In contrast, TM3 cells cultured with ECG exhibited significantly upregulated expression of these enzymes: StAR, 0.65 ± 0.13 (*p* = 0.0003); P450scc, 0.42 ± 0.03 (*p* = 0.0102); 3β–HSD, 0.51 ± 0.09 (*p* = 0.0009); CYP17a1, 0.53 ± 0.13 (*p* = 0.0008); and 17β–HSD, 0.52 ± 0.02 (*p* = 0.0001) (Figure 4E). To verify whether ECG had an effect on gene expression of testosterone metabolic enzymes in aged mice, qRT-PCR was used. The relative mRNA expression of *UGT2A3*, an enzyme associated with testosterone metabolism, was 0.14 ± 0.02 in the O-ECG group, which was significantly lower than that in the O-C group (*p* = 0.0005) (Figure 4F).

### 3.6. Target Prediction and Validation Results of Network Pharmacology

**Figure 5:**
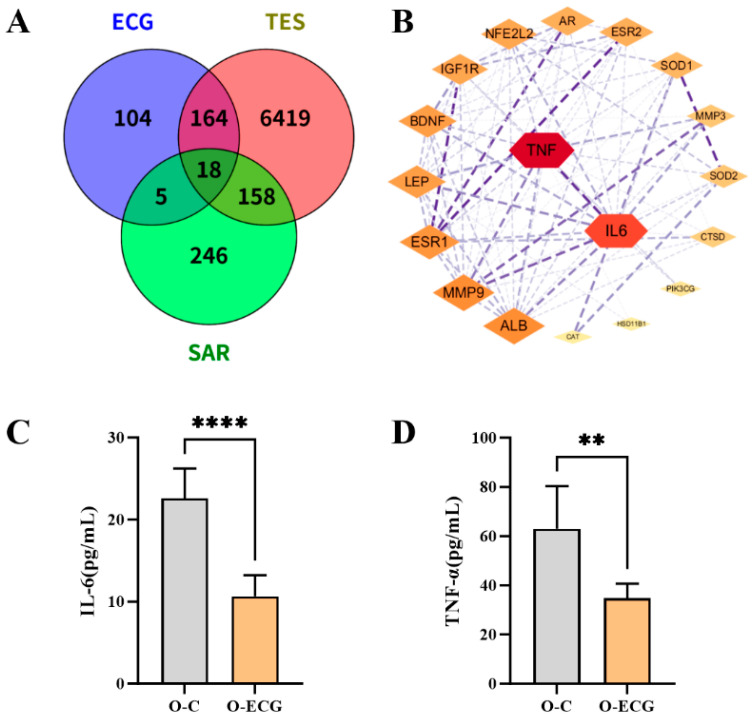
Network pharmacology target prediction and validation. (**A**) Venn diagram of target intersection genes. (**B**) PPI network diagrams of ECG, testosterone, and sarcopenia. (**C**,**D**) Concentrations of pro-inflammatory factors in mouse serum. Data are expressed as mean ± standard deviation (*n* = 6). Compared to the O-C group, ** *p* < 0.01, **** *p* < 0.0001.

After thorough searching and analysis, a total of 291 ECG compound targets were identified. These included 15 from the SwissTargetPrediction database, 153 from the PharmMapper database, 88 from the TargetNet database, 1 from the TCMSP database, and 68 from the CTD database. Additionally, a total of 6759 testosterone compound targets were identified, comprising 3 from the TCMSP database, 15 from the SwissTargetPrediction database, and 6753 from the CTD database. Furthermore, a total of 427 targets related to sarcopenia disease were obtained from the GeneCards database (Figure 5A). After intersecting the three results, the Wayne graph and PPI network graph were generated using Venny, the String database tools, and Cytoscape 3.10.2 software. From the results, the network pharmacology analysis revealed the key mediating roles of IL-6 and TNF-α in the regulatory network of ECG, testosterone, and sarcopenia (Figure 5B). Animal experimental data demonstrated significant alterations in serum inflammatory factor levels in the O-ECG group. Specifically, the serum IL-6 concentration in O-ECG mice (10.63 ± 2.60 pg/mL) was reduced to 47% of that in the O-C group (22.61 ± 3.63 pg/mL, *p* = 0.0001). Similarly, the serum TNF-α level in the O-ECG group (34.83 ± 5.87 pg/mL) decreased to 55.3% of the O-C group (63.03 ± 17.31 pg/mL, *p* = 0.0036) (Figure 5C,D).

## 4. Discussion

Catechins, a natural active polyphenol found in tea, comprise 12% to 24% of the dry weight of tea and are known to alleviate apoptosis [19] and delay muscle aging [14]. Additionally, natural polyphenols can enhance the number of skeletal muscle capillaries, promote the biosynthesis of mitochondria within muscle fibers [21], and increase the cross-sectional area of muscle fibers by slowing the rate of protein degradation associated with muscle atrophy [22]. This may explain why ECG in this experiment contributes to increased muscle mass and promotes a larger cross-sectional area of muscle fibers.

Behavioral experiments are significantly related to muscle anti-fatigue and motor ability. Grip strength evaluation is one of the most widely utilized methods. Treadmill locomotion is regarded as a measure of overall muscle function. Rod fatigue experiments are employed to assess the coordination of movement in animals. This experiment demonstrated that in vivo ECG supplementation could increase the absolute grip strength and relative grip strength of aged mice by 38.8% and 42.1%, respectively, and increase the running time and distance of the mice by 26.6% and 47.5%, respectively, indicating that ECG could significantly enhance the muscle endurance and muscle strength of aged mice (Figure 1B,E). It has been established that phenolic compounds, such as naringenin, can improve the motility of mice by enhancing oxidase activity, oxygen uptake, and PGC-1α expression, thereby augmenting endurance and exercise capacity [23]. Additionally, purple sweet potato anthocyanins enhance the body’s anti-fatigue and exercise capabilities by increasing the activity of skeletal muscle antioxidant enzymes and cell membrane enzymes [24]. Similarly, resveratrol improves anti-fatigue and exercise performance through the enhancement of skeletal muscle antioxidant enzyme activity and cell membrane enzyme activity. Furthermore, the expression of genes associated with synaptic function promotes synaptic vesicle circulation in the skeletal muscles of elderly rats [25], suggesting that ECG may operate through analogous regulatory mechanisms to enhance skeletal muscle motor performance.

Skeletal muscle mass is regarded as a critical indicator for assessing skeletal muscle atrophy [26]. In the present study, ECG supplementation significantly increased skeletal muscle CSA, body weight, skeletal muscle volume, and skeletal muscle index in aged mice compared with the control group, indicating that ECG intervention can significantly improve muscle atrophy in aged mice. As we age, the reduction in muscle mass is manifested through a decrease in the number of muscle fibers or the size of the cross-sectional area of muscle fibers, ultimately leading to muscle fiber atrophy [27]. Hematoxylin and eosin staining results showed that the cross-sectional area and number of muscle fibers in aged mice in the O-C group were significantly decreased, while ECG intervention increased the cross-sectional area and number of muscle fibers in aged mice by 55.2% and 41.4%, respectively (Figure 2D,E).

Skeletal muscle is a highly heterogeneous tissue composed of muscle fibers, each of which is proliferated, differentiated, and fused by myoblasts. Muscle progenitor cells (MPCs) are derived from dermal sarcoma and subsequently enter the myogenesis process under the regulation of *Myf5* and *Mrf4* [28]. The development of skeletal muscle is contingent upon the expression of myogenic regulators, with *MYOG*, *MYOD*, *MyHC*, and others collectively governing this process [29]. Myotubules continue to elongate and fuse to repair damaged myofibers or to form new multinucleated myofibers. Creatine kinase, encoded by the *MCK* gene, is widely expressed in skeletal muscle and serves as a significant marker of skeletal muscle development and differentiation [30]. In this study, qRT-PCR detection revealed that the relative expression levels of mRNA for skeletal muscle differentiation marker genes *MyoD*, *MyoG*, *MRF4*, and *MCK* in ECG-treated elderly mice increased significantly (Figure 2F). This finding demonstrates that the dietary addition of ECG may enhance the potential for skeletal muscle hyperplasia in elderly rats. Recent studies have indicated that natural polyphenols can promote muscle repair and regeneration by enhancing muscle differentiation. Catechins can directly promote the differentiation of myoblasts by upregulating the expression of myogenic regulators (MyoD, MyoG, Myf5) and myogenesis enhancers (MEF2) [17,31]. Additionally, they can indirectly support bone health by inhibiting the activity associated with muscle development [31].

Glucuronosyltransferases (UGTs) catalyze the transfer of glucuronic acid from uridine diphosphate glucuronic acid (UDPGA) to various endogenous substances, including bilirubin, steroid hormones, and fat-soluble vitamins, resulting in the formation of the corresponding glucuronide products [32]. Glucuronylation enhances water solubility and reduces the activity of the resulting products, thereby facilitating the clearance of metabolites through the kidneys, bile, or intestines. This process plays a crucial regulatory role in maintaining the balance of endogenous substances and in the elimination of exogenous substances from the body. The UGT family is primarily studied in the context of drug metabolism, but it also significantly contributes to the metabolism of toxic carcinogens and the breakdown of endogenous carcinogenic molecules in tumors [33]. For instance, bladder cancer and prostate cancer exhibit a deficiency in UGTs, which impairs androgen metabolism, consequently promoting the onset and progression of cancer [34].

*UGT2A3* (UDP-glucuronosyltransferase 2A3) is a protein belonging to the UGT family. Transcriptomic analyses revealed that *UGT2A3* is the most significantly downregulated gene among the major enrichment signal pathways of differentially expressed genes (Figure 3E) and is involved in the metabolic process of testosterone within the steroid hormone biosynthesis pathway. After testosterone exerts its effects on mouse skeletal muscle, it may be directly converted into testosterone glucuronide by the UGT2A3 enzyme, or it may be transformed into estradiol-17β through the action of the CYP19A enzyme. Estradiol-17β can then be directly converted into estradiol-17β-3-glucuronide by the UGT2A3 enzyme, or it may be further metabolized into estriol via the CYP1A1 enzyme. Subsequently, estriol can also be converted by the UGT2A3 enzyme into 16-glucuronidine triol. The metabolic enzyme UGT2A3 plays a crucial role in testosterone metabolism. In mice administered ECG over an extended period, there was a highly significant reduction in the expression of the UGT2A3 gene in skeletal muscle (Figure 4F), resulting in sustained high levels of testosterone in the gastrocnemius muscle (Figure 4C). This phenomenon can regulate the expression of differentiation-related genes in skeletal muscle, leading to increased muscle fiber size and delayed muscle loss.

Testosterone is a crucial pleiotropic sex hormone that significantly contributes to muscle growth and maintenance in both men and women [35]. It reduces myocyte protein degradation by supporting myocyte metabolism and inhibiting the ubiquitin-proteasome pathway. Additionally, testosterone promotes skeletal muscle protein synthesis [36,37] and increases the number of muscle satellite cells, thereby facilitating muscle regeneration and repair [10]. Testosterone deficiency reduces the phosphorylation levels of the mammalian target of rapamycin (mTOR) and impairs Akt activation, which downregulates protein synthesis and results in a decrease in skeletal muscle mass [38]. Testosterone is primarily synthesized and secreted by testicular interstitial cells, with its synthesis regulated by the hypothalamus–pituitary–testis axis (HPT), transcription factors, and coenzyme factors. Cholesterol serves as the raw material for testosterone synthesis in these interstitial cells. The steroidogenic acute regulatory [39] protein, located on the outer membrane of mitochondria, facilitates the transport of cholesterol to the inner mitochondrial membrane [40]. Once there, cholesterol side-chain cleavage cytochrome P450scc catalyzes the conversion of cholesterol into pregnenolone. Following its transport to the endoplasmic reticulum, pregnenolone is enzymatically converted into progesterone by 3β-hydroxysteroid dehydrogenase (3β-HSD). Subsequently, both progesterone and pregnenolone undergo multiple enzymatic transformations via CYP17A1, leading to the formation of androstenedione. Finally, androstenedione is converted into testosterone through the action of 17β-hydroxysteroid dehydrogenase (17β-HSD) [41]. The rate-limiting step of this process significantly influences testosterone synthesis and secretion.

In this cell experiment, treatment of TM3 cells with 50 μg/mL ECG significantly increased the testosterone content and the expression of testosterone synthetase genes, including *StAR*, *P450scc*, *3β–HSD*, *CYP17a1*, and *17β–HSD*, as well as their corresponding protein levels in TM3 cells (Figure 4D,E). These findings suggest that appropriate concentrations of ECG can enhance testosterone synthesis by upregulating the expression of genes related to testosterone synthesis enzymes. Interestingly, the levels of muscle steroid hormones were significantly correlated with muscle strength and CSA [42]. This experiment demonstrated that testosterone levels in the gastrocnemius muscle of mice administered ECG significantly increased (Figure 4C). The exogenous supplementation of ECG appears to enhance the levels of skeletal muscle steroid hormones and may contribute, to some extent, to increases in muscle strength and CSA. Related studies have demonstrated that supplementing with Qiang Juice Tablets can regulate the expression of CYP11A1 and CYP17A1, thereby alleviating the compensatory expression of StAR and 3β-HSD in rat testicles. This regulation helps restore and maintain testosterone secretion levels to normal in elderly rats [43]. Furthermore, natural plant extracts have shown significant potential in enhancing the viability of testicular interstitial cells [44]. Both in vitro and in vivo tests indicate that catechins promote testosterone secretion in rats by increasing the enzyme activity of 17β-HSD [45]. This study further confirmed that ECG upregulates the expression of genes related to testosterone synthesis, thereby enhancing testosterone secretion and influencing the differentiation of skeletal muscles in male mice. However, female individuals lack testicular interstitial cells and possess a limited capacity to synthesize testosterone. Consequently, it remains to be verified whether ECG produces a similar effect in elderly females.

Network pharmacology can establish a network of interactions between drugs and targets, based on the multi-component and multi-target characteristics of natural materials. This approach enables a comprehensive analysis of the network, revealing potential drug-effective components and targets for disease treatment, as well as mechanisms of action that provide important references for screening active ingredients and understanding drug mechanisms. Furthermore, network pharmacological analysis indicates that ECG plays a significant role in regulating cytokines. In this experiment, the serum proinflammatory factor content in mice administered ECG was significantly reduced (Figure 5C,D), indicating that ECG can regulate cytokines, promote steroid hormone synthesis, and subsequently modulate the expression of genes associated with skeletal muscle differentiation. Cytokines are synthesized and secreted by various histocellular cells and are primarily categorized into growth factors, interleukins, and interferons. Several cytokines play a role in regulating the synthesis of testosterone, with their mechanisms of action potentially linked to the condition of testicular interstitial cells. For instance, TNF-α and IL-6 exert significant inhibitory effects on steroid production [46]. NUR77 serves as a crucial regulator of multiple genes involved in steroid hormone production. Additionally, TNF-α influences this process by interfering with the nuclear receptor NUR77 [47]. In vitro studies have demonstrated that steroid hormone deficiency is correlated with elevated expression and secretion of IL-6 and TNF-α [48].

Finally, this study evaluated mouse motor capacity using a combination of limb grip strength tests, rotarod fatigue tests, and treadmill exercise tests, visually demonstrating the effects of ECG on muscular function. These findings were corroborated by transcriptomic analysis and in vitro cell experiments, which revealed that ECG ameliorates sarcopenia in aged mice by regulating testosterone synthesis and metabolism. Notably, due to the absence of Leydig cells in female individuals—resulting in significantly lower testosterone production capacity than males—it remains to be determined whether ECG exerts similar effects in aged females. This question warrants further investigation to validate potential gender-specific responses.

In addition, the accuracy of ECG dosage is also an important issue to be considered in the experiment. In this experimental protocol, the maximum dosage of ECG was set at 160 mg/kg/d, with the solution concentration reaching 16 mg/mL—exceeding its room-temperature solubility of 5 mg/mL. To address this, we applied short-term ultrasonic treatment to facilitate dissolution. Notably, as a polyphenolic antioxidant, ECG is highly sensitive to thermal and oxidative degradation. Uncontrolled temperature during processing could significantly reduce its antioxidant activity, thereby compromising the compound’s intended efficacy. To avoid the increase in temperature during the ultrasound process that may affect the ECG activity, we recommend future studies maintain a low-temperature environment by adding ice cubes to the ultrasonic solution, thus preserving ECG’s biological activity to the greatest extent. Additionally, while the actual gavage dosage for mice was lower than the theoretical value due to solubility constraints, we ensured consistent total ECG intake across all subjects. The findings from this study are expected to provide valuable references for similar research.

## 5. Conclusions

**Figure 6:**
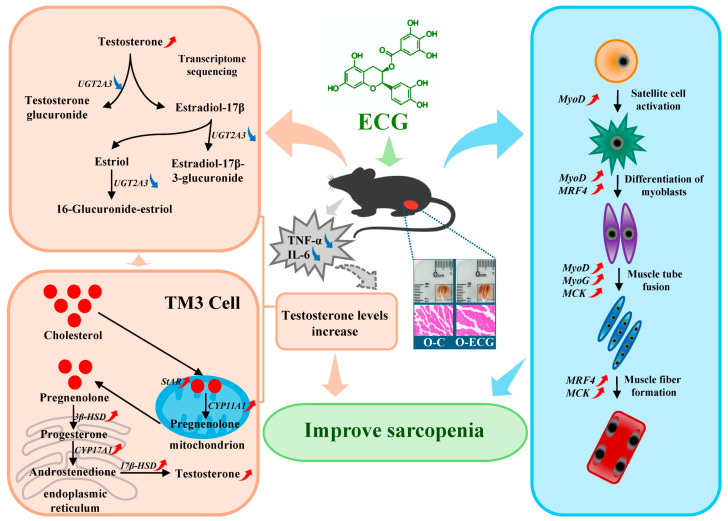
Mechanisms of ECG to improve sarcopenia of skeletal muscle.

This study employed aged C57BL/6J mice as models and integrated phenotypic analysis, histomorphological observation, transcriptomic sequencing, and network pharmacology target prediction to investigate the ameliorative effects of ECG on age-related sarcopenia. Our findings indicate that ECG exerts dual regulatory effects on testosterone metabolism: (1) upregulating the expression of genes and proteins involved in testosterone biosynthesis, including StAR, P450scc, 3β–HSD, CYP17a1, and 17β–HSD; and (2) downregulating the expression of Ugt2A3, a key enzyme in testosterone catabolism, thereby reducing metabolic clearance in skeletal muscle. Through this bidirectional regulation, ECG effectively modulates skeletal muscle differentiation and mitigates sarcopenia in aged mice. Additionally, ECG enhances cytokine signaling pathways, further elevating testosterone levels and promoting myogenic differentiation (Figure 6). Collectively, these results suggest that ECG—a natural polyphenol—may delay skeletal muscle aging and protect against age-related declines in muscle function via dietary supplementation.

## Data Availability

The original contributions presented in this study are included in the article. Further inquiries can be directed to the corresponding author.

## Supplementary Materials

The following supporting information can be downloaded at: https://www.mdpi.com/article/10.3390/foods14142495/s1, Table S1: Primer sequences; Table S2: The impact on organ indices in each group of mice.

## Abbreviations

The following abbreviations are used in this manuscript:

SkM	skeletal muscle
ECG	epicatechin gallate
TM3	Leydig cells
T	Testosterone
IGF-I	insulin-like growth factor-1
H&E	hematoxylin and eosin
TEM	transmission electron microscopy
CSA	cross-sectional area
DEGs	differentially expressed genes
PPI	protein–protein interaction
TNF-α	tumor necrosis factor-α
IL-6	interleukin-6
MPCs	muscle progenitor cells
UGTs	glucuronosyltransferases
UDPGA	uridine diphosphate glucuronic acid
mTOR	mammalian target of rapamycin
HPT	hypothalamus pituitary testis
