# Supplementary material for: Epicatechin Gallate Regulation of Steroid Hormone Levels Improves Sarcopenia in C57BL/6J Mice
Source: Foods. 2025 Jul 16;14(14):2495. doi: 10.3390/foods14142495 (PMC12295562; doi:10.3390/foods14142495)
Supplement: Supplementary file 1 [file foods-14-02495-s001.zip › foods-3698401-supplementary.pdf]

Epicatechin gallate regulation of steroid hormone levels improves sarcopenia in C57BL/6J mice

Zi-wei Huang[a,b,c,d], Mei-feng Liu[b,c,d], Yu-fei Zhou[b,c,d], Yi-yu Tang[b,c,d], Jian-an Huang[b,c,d], Sheng Zhang[b,c,d,*], Zhong-hua Liu [b,c,d,*], Ai-ling Liu [a,b,c,d,*]

[a] *College of Bioscience and Biotechnology, Hunan Agricultural University, Changsha 410128, Hunan, China*

[b] *National Research Center of Engineering and Technology for Utilization of Botanical Functional Ingredients, Changsha 410128, Hunan, China*

[c] *Key Laboratory of Tea Science of Ministry of Education, Hunan Agricultural University, Changsha 410128, Hunan, China;*

[d] *Yuelushan Laboratory, Changsha 410128, Hunan, China*

***Corresponding authors:**

Ai-ling Liu, College of Bioscience and Biotechnology, Hunan Agricultural University, Changsha 410128, Hunan, China; Email: liuailing@hunau.edu.cn

Zhong-hua Liu, National Research Center of Engineering and Technology for Utilization of Botanical Functional Ingredients, Changsha 410128, Hunan, China; Email: liuzhonghua@hunau.edu.cn

Sheng Zhang, National Research Center of Engineering and Technology for Utilization of Botanical Functional Ingredients, Changsha 410128, Hunan, China; Email: zhangsheng@hunau.edu.cn

Supporting Information

Methods

Organ Index. Following blood collection, each mouse was euthanized via cervical dislocation, and the spleen, thymus, heart, kidneys, and liver were excised. Excess adipose tissue was carefully removed, and the organs were rinsed with saline to eliminate surface blood. The organs were then gently blotted with filter paper to remove any excess moisture, weighed, and the organ index was calculated. The formula for calculating the organ index is as follows:

Organ index = Organ weight (g) / Body weight (g) × 100%

Supporting Table S1. Primer Sequences.

Supporting Table S2. The impact on organ indices in each group of mice.

Supporting Table S1. Primer Sequences.

primer		5′ to 3′
β-Actin	F	TCACCAACTGGGACGACATG
	R	GTCACCGGAGTCCATCACGAT
StAR	F	TCTACCACCACCTCCAAGCGAAA
	R	TGGGCATACTCAACAACCAGGAA
P450scc	F	AGGTCCTTCAATGAGATCCCTT
	R	TCCCTGTAAATGGGGCCATAC
3β–HSD	F	TGCAGACAAAGACCAAGGTG
	R	ACAGCAGCAGTGTGGATGAC
CYP17a1	F	GCCCAAGTCAAAGACACCTAAT
	R	GTACCCAGGCGAAGAGAATAGA
17β–HSD	F	GATGTGGCTGTCAACTGTGC
	R	TTGATAACCCGCTGGAAGTC
MyOD	F	CCATCCGCTACATCGAAGGT
	R	GTAGTAGGCGGTGTCGTAGC
MyoG	F	ATCTGCACTCCCTTACGTCC
	R	GACAGCCCCACTTAAAAGCC
MRF4	F	AACTACATTGAGCGTCTGC
	R	AGAATTTCTTGCTTGGGTTTG
MCK	F	TAGTCACACCCTGTAGGCTCCTCTAT
	R	ATTTCTCTCAGTCCCTACCTGGCT
UGT2A3	F	CGAAGCTGTGGGAAACTTCCA
	R	GGGCCTTCCTAATGCCTTACT

Supporting Table S2. The impact on organ indices in each group of mice.

group	Cardiac Index	Spleen Index	Thymus Index	Liver index	Kidney index
Y-C	0.47±0.03	0.28±0.05	0.11±0.01	3.17±0.46	1.09±0.09
Y-ECG	0.46±0.03	0.28±0.12	0.10±0.01	3.15±0.43	1.09±0.13
M-C	0.44±0.02	0.23±0.02	0.09±0.03	3.07±0.08	1.02±0.05
M-ECG	0.45±0.04	0.24±0.07	0.09±0.02	3.06±0.37	1.04±0.14
O-C	0.52±0.07	0.23±0.03	0.07±0.02	3.19±0.05	1.10±0.02
O-ECG	0.53±0.03	0.25±0.02	0.07±0.01	3.18±0.18	1.12±0.04
